# Stepwise Lengthening of the Quadriceps Extensor Mechanism for Severe Obligatory and Fixed Patella Dislocators

**DOI:** 10.1016/j.eats.2021.01.034

**Published:** 2021-04-18

**Authors:** Bridget Ellsworth, Sofia Hidalgo Perea, Daniel W. Green

**Affiliations:** Department of Orthopaedic Surgery, Hospital for Special Surgery, New York, New York, U.S.A.

## Abstract

We introduce an algorithm of independently performing vastus lateralis lengthening followed by Z lengthening of the rectus and intermedius portion of the quadriceps tendon to treat fixed and obligatory patellar instability in the pediatric population. Performing this procedure in conjunction with medial patellofemoral ligament reconstruction minimizes subsequent episodes of instability without creating extensor mechanism weakness or contracture.

Medial patellofemoral ligament (MPFL) reconstruction is the standard of care to treat patellofemoral instability in the pediatric population and has been extensively described.^1^ However, MPFL reconstruction alone is typically not sufficient to remove the deforming forces on the patella in cases of fixed or obligatory lateral patellar dislocation in flexion.^2^ Obligatory dislocation is defined as dislocation with every episode of knee flexion with auto-reduction on extension. Fixed dislocation refers to an irreducible lateral patella regardless of knee position. Obligatory and fixed dislocators have tight lateral structures and a shortened extensor mechanism that contribute to lateral instability (Fig 1).

**Fig 1:**
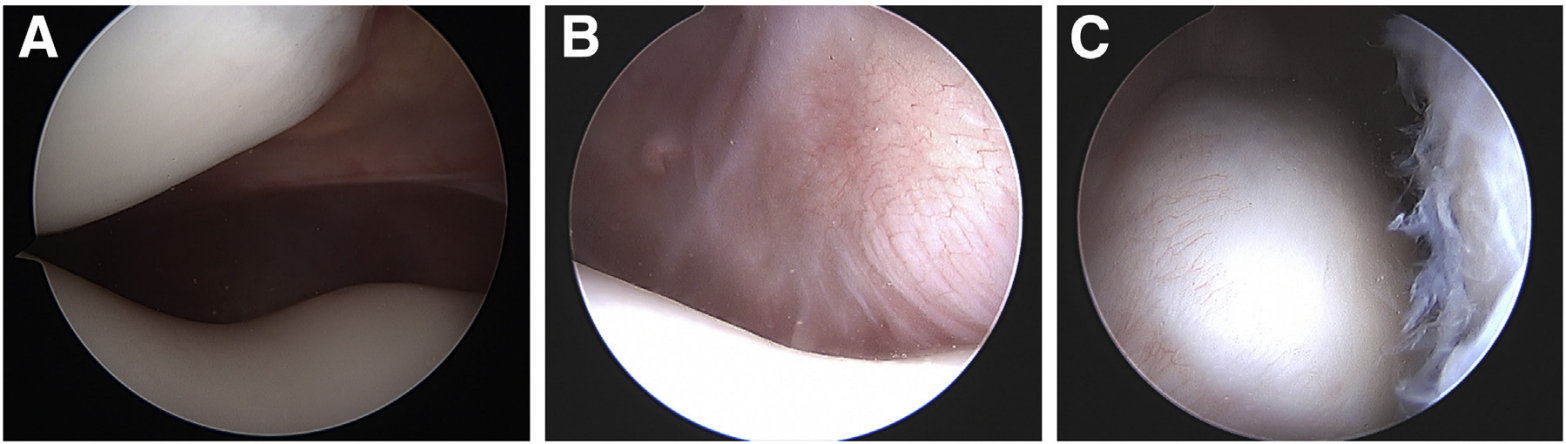
Arthroscopic images of pediatric obligatory (A) and fixed (B, C) dislocators prior to stepwise lengthening of quadriceps extensor mechanism and medial patellofemoral ligament reconstruction. Both patients underwent right-knee arthroscopy, and all images were taken with the knee in extension from the anterolateral portal. (A) Lateral patellar tilt and subluxation. (B, C) Empty hypoplastic trochlea (B) with patella dislocated in lateral gutter (C).

We believe that in cases of severe patellar instability, the vastus lateralis is typically more contracted than the rectus, vastus intermedius, or vastus medialis oblique (VMO). We also believe that the contracted nature of the lateral quadriceps provides a lateral vector of force on the patella.

We believe that a stepwise approach that is characterized by extensive lateral release or lengthening, vastus lateralis lengthening, and finally (if needed), separate Z lengthening of the rectus and intermedius tendon is superior to V-Y lengthening because it permits the surgeon to preferentially lengthen the lateral aspect of the quadriceps tendon more than the medial aspect. This article will review our stepwise approach. Components of this approach have been previously published, including the lateral retinacular lengthening and vastus lateralis lengthening procedures.^3^, ^4^, ^5^

We describe a stepwise lengthening technique for the extensor mechanism in which the lateral retinaculum is lengthened followed by the vastus lateralis tendon (Fig 2). The main quadriceps tendon then undergoes Z lengthening only if the patella continues to dislocate in flexion after lateral retinacular and vastus lateralis lengthening. The main benefit of the technique is that the lateral structures are lengthened first, which decreases the lateral pull on the patella from the quadriceps mechanism. By use of this algorithm, formal Z lengthening of the quadriceps tendon is not required in about half of the cases performed by the senior author (D.W.G.). If Z lengthening is required, the superomedial aspect of the quadriceps tendon is left intact. The vastus medialis is also left intact. This creates an overall medial vector of pull on the patella from the quadriceps mechanism, thus facilitating patellar tracking in cases of lateral patellar dislocation. MPFL reconstruction is then performed.

**Fig 2:**
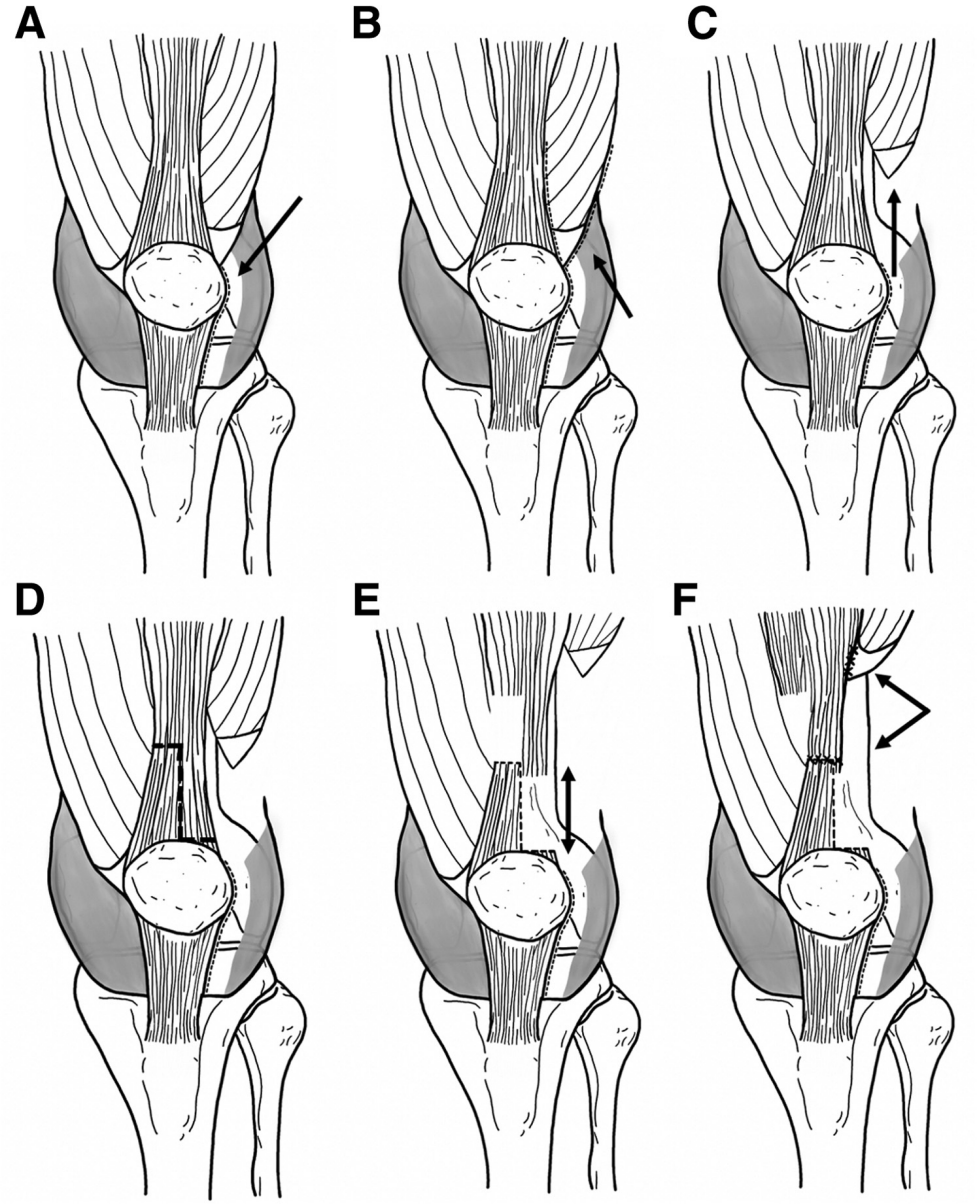
Stepwise lengthening of extensor mechanism to treat patients with obligatory or fixed patellar dislocations, shown in left knee. (A) A lateral retinacular release is performed as indicated by the arrow. (B, C) The vastus lateralis tendon is identified and the distal aspect is released from the superolateral patella, the adjacent quadriceps tendon, and the lateral synovial bands and lateral intermuscular septum (B) so that it can be completely mobilized proximally (see arrow) (C). If Z lengthening is indicated, a Z cut of the quadriceps tendon is performed. The distal aspect of the Z is at the superolateral aspect of the patella and spans half the transverse width of the quadriceps tendon, leaving the medial quadriceps tendon attached to the superomedial patella. (D) The longitudinal aspect of the Z is in the center of the tendon, and the proximal aspect of the Z is medial. (E) The tendon is typically lengthened about 2 cm (see arrow). (F) The quadriceps tendon is repaired, and the distal aspect of the vastus lateralis is reattached to the lateral aspect of the main quadriceps tendon, proximal to the patella (see arrow).

## Technique

The patient is positioned supine on the operating table with a nonsterile tourniquet placed on the thigh and a bump placed under the ipsilateral hip so that the patella is pointing to the ceiling. A midline longitudinal skin incision is made from proximal to the patella to the joint line. Dissection is carried through subcutaneous tissue with electrocautery until the patella and quadriceps tendon are visualized. Full-thickness skin flaps are developed to allow visualization of the lateral aspect of the patella, the vastus lateralis tendon, and the quadriceps tendon.

**Fig 3:**
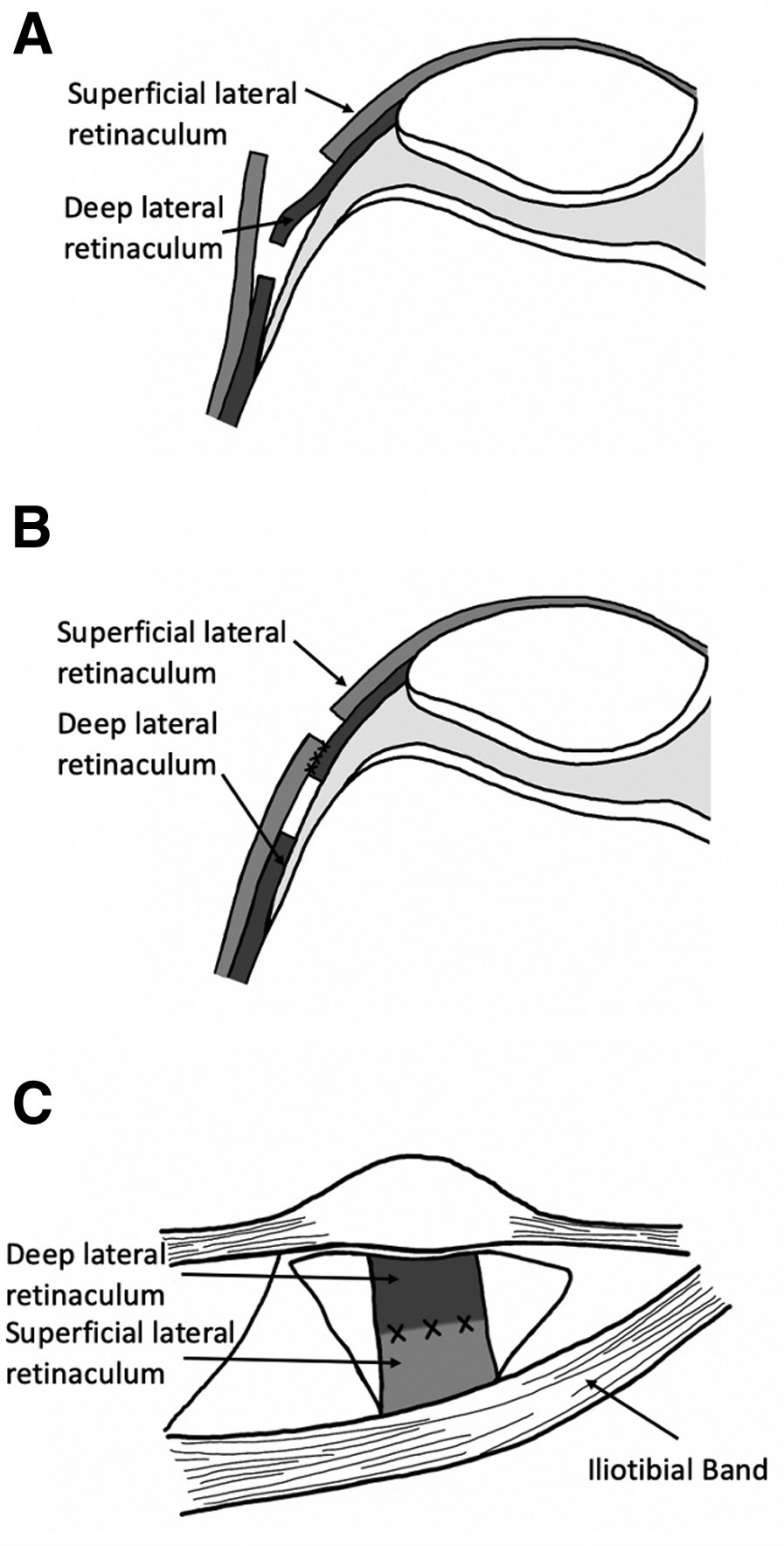
Lateral retinacular lengthening in right knee: axial view of knee in extension (A, B) and sagittal view (C). (A) An interval is sharply created between the superficial oblique and deep transverse retinaculum just lateral to the patella. The superficial oblique ligament is peeled posteriorly. The deep transverse retinaculum is then followed posteriorly, transected at the level of the iliotibial band, and elevated off the underlying capsule. (B, C) The lateral retinaculum is repaired at the level of the patella in a lengthened position.

First, an extensive lateral retinacular release is performed as previously described by Larson et al.^3^ to allow for lateral retinacular lengthening at the end of the case (Video 1, Fig 3). The release extends distally just lateral to the patellar tendon to the joint line. An interval is sharply created between the superficial oblique and deep transverse retinaculum just lateral to the patella. The superficial oblique ligament is peeled posteriorly. The deep transverse retinaculum is then followed posteriorly, transected at the level of the iliotibial band, and elevated off the underlying capsule. The lateral capsule is released, and the lateral retinaculum is repaired at the level of the patella in a lengthened position at the end of the case. Lateral lengthening as opposed to release provides further stability postoperatively. However, it is not always possible to repair the lateral retinaculum in knees with severe contracture of the lateral tissues.

The vastus lateralis tendon is then identified as it inserts into the superolateral aspect of the patella, as well as into the main quadriceps tendon. First, it is released from the superolateral aspect of the patella using electrocautery. Second, it is released from the adjacent quadriceps tendon about 5 cm proximal to the patella. Third, the tendon is released from the remaining lateral synovial bands and lateral intermuscular septum that are tethering it so that it can be completely mobilized proximally.

The knee is taken through a full range of motion. Spontaneous dislocation of the patella in flexion after the vastus lateralis has been released is an indication that the quadriceps tendon is too tight and formal lengthening of the main quadriceps tendon (consisting of the rectus femoris and vastus intermedius tendons) is required. Z lengthening is required in about half of obligatory dislocators treated by the senior author. The distal aspect of the “Z” cut is made at the superolateral aspect of the patella and spans half of the transverse width of the quadriceps tendon, leaving the medial quadriceps tendon attached to the superomedial patella. The longitudinal aspect of the Z is in the center of the tendon and measures about 3 to 4 cm in length. The proximal aspect of the Z exits the quadriceps tendon medially. The vastus medialis does not typically need to be lengthened because it has been functionally lengthened by the previous lateral patellar instability.

Repair of the quadriceps tendon is performed under tension at approximately 60° of knee flexion, and the tendon is typically lengthened about 2 cm. A multiple–interrupted suture repair of the tendon with nonabsorbable suture (FiberWire [Arthrex] or Ethibond [Johnson & Johnson]) is performed and augmented with several interrupted absorbable sutures (No. 0 or 1 Vicryl; Johnson & Johnson). The knee is then flexed to ensure that the patella does not continue to spontaneously dislocate in flexion after quadriceps lengthening.

The vastus lateralis is reattached to the main quadriceps tendon about 4 to 6 cm proximal to the patella using a similar multiple–interrupted suture repair to the quadriceps Z lengthening. At the conclusion of the repair, the vastus lateralis tendon is always lengthened more than the rectus and intermedius tendons.

The MPFL is reconstructed after the quadriceps lengthening. Prior to closure, range of motion with flexion to at least 110° is confirmed to ensure that the quadriceps repair remains intact and is not under excess tension. However, this is a balance because there should be some quadriceps tension with flexion to ensure that an extensor lag does not develop. The patella should be observed tracking in the trochlea for several cycles of flexion and extension.

After surgery, the patient is placed in a hinged knee brace that is locked in extension for ambulation for the first 4 to 6 weeks. Weight bearing is allowed as tolerated with crutches for support. The brace is unlocked when the patient is not ambulating, and range of motion is started immediately with the goal of achieving 90° of flexion in the first 4 weeks postoperatively. Formal physical therapy starts at week 2. After week 6, the patient is allowed to ambulate without a brace once the quadriceps function has returned. He or she continues to work on regaining range of motion and quadriceps strength with closed-chain exercises. Functional progression to running and jumping is typically initiated after 4 months postoperatively.

## Discussion

Fixed and obligatory cases of patellofemoral instability are difficult to manage, and few techniques are published to guide treatment. We describe a technique of stepwise lengthening of the quadriceps mechanism in cases of severe (fixed or obligatory) patellofemoral instability. By releasing the vastus lateralis tendon and reattaching it proximally to the quadriceps tendon, it is not only lengthened but the lateral vector of force generated by the vastus lateralis is shifted medially. This realignment decreases the amount of lateral dislocating force on the patella. Similarly, the vastus medialis tendon is not lengthened in this technique because it generates a medial vector of force on the patella, which may facilitate patellar stability. Additionally, the vastus medialis is likely already functionally lengthened as a result of previous patellar dislocations.

Other quadriceps lengthening techniques have been described: Martin et al.^6^ reported on a technique involving oblique transection of the main quadriceps tendon from distal-medial to proximal-lateral and the transposition of the distal portion medially relative to the proximal portion to facilitate patellar stability. The V-Y quadricepsplasty technique, involving dissection of both the vastus medialis and vastus lateralis from the main quadriceps tendon, was published by Curtis and Fisher^7^ in 1969 to facilitate reduction of congenital knee dislocations. Congenital knee dislocation involves hyperextension, subluxation, or dislocation of the tibia on the femur due to contracture and fibrosis of the entire extensor mechanism.^7^ Thus, it is reasonable to use a V-Y quadricepsplasty or a modification to lengthen both the medial and lateral aspects of the quadriceps tendon equally in severe cases of congenital knee dislocation. However, V-Y quadricepsplasty has been associated with extensor lag and quadriceps weakness.^8^ As previously discussed, cases of obligatory and fixed patellar dislocation involve contracture of the lateral extensor mechanism. Thus, we believe it is more logical in these cases to use a technique that preferentially lengthens the lateral extensor mechanism while leaving the medial quadriceps intact.

Fortunately, patients with severe obligatory and fixed patellar dislocations are relatively rarely encountered. However, the low incidence of this condition makes it difficult to develop evidence-based treatment algorithms, and most published literature consists of retrospective studies. Two Level IV studies have been published recently describing the results of surgical treatments for obligatory or fixed patellar instability in skeletally immature patients. These treatments differ from the technique outlined in this article. In the first study, published in 2019, Sever et al.^9^ described the results of 12 patients (15 knees) after extensive subperiosteal quadriceps realignment and soft-tissue medial plication along with distal realignment using the Roux-Goldthwait procedure. Eleven patients had no recurrence of instability at a mean follow-up of 46 months, and all patients had improved active knee range of motion after surgery.

In the second study, published in 2020, Danino et al.^10^ described the results of 34 patients (46 knees) after a “4-in-1” procedure including a combination of the Roux-Goldthwait procedure, VMO advancement, lateral release, and the Galeazzi procedure in all patients. The mean Kujala score was 93 (range, 83-100), and 18% of patients had recurrent instability at a mean follow-up of 52 months. Long-term bracing was required in 6 knees.

Notably, neither of the aforementioned studies used MPFL reconstruction to stabilize the patella; instead, they used more extensive quadriceps dissection with realignment, as well as distal realignment and advancement of the VMO.^9^^,^^10^ Neither study reported on the incidence or severity of extensor lag after these procedures. However, both studies reported good objective and subjective outcomes, which shows that there may be multiple useful techniques for treating this challenging problem. We prefer the technique outlined in this article because we believe it provides good patient outcomes while minimizing quadriceps dissection and obviating distal realignment. Table 1 presents pearls and pitfalls of our technique.

**Table 1:** Pearls and Pitfalls of Stepwise Lengthening of Quadriceps Extensor Mechanism for Severe Obligatory and Fixed Patella Dislocators

Pearls
Lateral retinacular lengthening as opposed to release may improve patellar stability.
The surgeon should lengthen the lateral retinaculum followed by the vastus lateralis. If the patella continues to dislocate in flexion, Z lengthening of the main quadriceps tendon should be performed. When performing Z lengthening of the main quadriceps tendon, the surgeon should leave the medial aspect of the quadriceps tendon attached to the patella and should not lengthen the vastus medialis.
Pitfalls
The surgeon should make sure to release the vastus lateralis completely from the synovial bands and lateral intermuscular septum to allow for proximal mobilization.
The surgeon should make sure to check range of motion of the knee prior to closure to ensure patellar tracking and to ensure that the quadriceps repair is not under too much or too little tension.

The described technique is safe because there are no major neurovascular structures at risk. Potential complications of the technique include quadriceps weakness, extensor lag, and recurrent patellar instability, in addition to the complications of MPFL reconstruction. However, residual extensor lag has not been observed in the 24 patients we have treated with this approach.

## Supplementary Data

Video 1 A case of obligatory patellar dislocation with knee flexion in an 8-year-old boy with nail-patella syndrome. The steps of the procedure include lateral retinacular release or lengthening, vastus lateralis lengthening, Z lengthening of the quadriceps tendon, and medial patellofemoral ligament reconstruction. Of note, the surgical procedure is shown in a right knee. Lateral retinacular lengthening is performed by sharply creating an interval between the superficial oblique and deep transverse retinaculum just lateral to the patella. The superficial oblique ligament is peeled posteriorly. The deep transverse retinaculum is then followed posteriorly, transected at the level of the iliotibial band, and elevated off the underlying capsule. The lateral capsule is released, and the lateral retinaculum is repaired at the level of the patella in a lengthened position at the end of the case. Next, the vastus lateralis is lengthened. First, it is released from the superolateral aspect of the patella using electrocautery. Second, it is released from the adjacent quadriceps tendon about 5 cm proximal to the patella. Third, the tendon is released from the remaining lateral synovial bands and lateral intermuscular septum that are tethering it so that it can be completely mobilized proximally. The knee is then taken through a range of motion. If the patella continues to dislocate in flexion, this is an indication that the quadriceps tendon is too tight. Z lengthening of the main quadriceps tendon (consisting of the rectus femoris and vastus intermedius tendons) is then performed, leaving the medial quadriceps tendon attached to the superomedial patella. The vastus lateralis is reattached to the main quadriceps tendon about 4 to 6 cm proximal to the patella using a multiple–interrupted suture repair. This is followed by medial patellofemoral ligament reconstruction.

ICMJE author disclosure forms
